# DNA Methylation Signatures in Paired Placenta and Umbilical Cord Samples: Relationship with Maternal Pregestational Body Mass Index and Offspring Metabolic Outcomes

**DOI:** 10.3390/biomedicines12020301

**Published:** 2024-01-27

**Authors:** Ariadna Gómez-Vilarrubla, Berta Mas-Parés, Gemma Carreras-Badosa, Alexandra Bonmatí-Santané, Jose-Maria Martínez-Calcerrada, Maria Niubó-Pallàs, Francis de Zegher, Lourdes Ibáñez, Abel López-Bermejo, Judit Bassols

**Affiliations:** 1 Maternal-Fetal Metabolic Research Group, Girona Institute for Biomedical Research (IDIBGI), 17190 Salt, Spain; 2 Pediatric Endocrinology Research Group, Girona Institute for Biomedical Research (IDIBGI), 17190 Salt, Spain; 3 Department of Gynecology, Dr. Josep Trueta Hospital, 17007 Girona, Spain; 4 Department of Development & Regeneration, University of Leuven, 3000 Leuven, Belgium; francis.dezegher@kuleuven.be; 5 Endocrinology, Pediatric Research Institute, Sant Joan de Déu Children’s Hospital, 08950 Esplugues de Llobregat, Spain; 6 Centro de Investigación Biomédica en Red de Diabetes y Enfermedades Metabólicas Asociadas (CIBERDEM), Health Institute Carlos III (ISCIII), 28029 Madrid, Spain; 7 Department of Pediatrics, Dr. Josep Trueta Hospital, 17007 Girona, Spain

**Keywords:** DNA methylation, placenta, umbilical cord, pregestational obesity

## Abstract

An epigenomic approach was used to study the impact of maternal pregestational body mass index (BMI) on the placenta and umbilical cord methylomes and their potential effect on the offspring’s metabolic phenotype. DNA methylome was assessed in 24 paired placenta and umbilical cord samples. The differentially methylated CpGs associated with maternal pregestational BMI were identified and the metabolic pathways and the potentially related diseases affected by their annotated genes were determined. Two top differentially methylated CpGs were studied in 90 additional samples and the relationship with the offspring’s metabolic phenotype was determined. The results showed that maternal pregestational BMI is associated with the methylation of genes involved in endocrine and developmental pathways with potential effects on type 2 diabetes and obesity. The methylation and expression of *HADHA* and *SLC2A8* genes in placenta and umbilical cord were related to several metabolic parameters in the offspring at 6 years (weight SDS, height SDS, BMI SDS, Δ BW-BMI SDS, FM SDS, waist, SBP, TG, HOMA-IR, perirenal fat; all *p* < 0.05). Our data suggest that epigenetic analysis in placenta and umbilical cord may be useful for identifying individual vulnerability to later metabolic diseases.

## 1. Introduction

Accumulating evidence suggests that overweight and obesity in children are influenced by an intrauterine environment [1]. This is known as fetal programming and implies that environmental events can reset the physiological development of the embryo and the fetus. The mechanisms underlying these effects might involve an impaired placental transfer of nutrients during fetal development, which may cause permanent adaptations in appetite, energy metabolism, and neuroendocrine function in the offspring, which predispose individuals to a greater risk of obesity and metabolic diseases in later life [2]. Among the wide range of in utero exposures, maternal obesity is a significant risk factor for cardiometabolic outcomes in the progeny [3]. Studies show a clear relationship between increased maternal pregestational BMI, increased adiposity levels, and adverse cardiometabolic profile in their offspring at school age [4,5,6]. Whether these associations reflect direct intrauterine causal mechanisms or are part of the obesity-inherited condition remains unclear.

The mechanisms underlying these associations are still unknown. However, mounting evidence from animal and human studies supports that in utero exposures are associated with epigenetic modifications, including DNA methylation, which can, in turn, alter the transcriptional capacity of genes involved in metabolism [7]. DNA methylation, the most commonly investigated epigenetic mechanism influencing transcriptional regulation, consists of the addition of a methyl group to the 5′ carbon of a cytosine followed by a guanine, resulting in a methylated CpG site (CpGs). The distribution and pattern of methylation are not uniform throughout the genome, as it occurs mainly in the so-called CpG islands (CGI), which correspond to regions that are longer than 200 bp, with a cytosine and guanine content of at least 50%, and a ratio of observed to expected CG higher than 0.6. It is estimated that 70% of the promoters contain CGIs. Unmethylated CGIs have been related to transcriptionally active genes, whereas methylated CGIs are related to transcriptional repression [8].

The relationship between epigenetic mechanisms (especially DNA methylation) and obesity is emerging as a new field with potential clinical implications [9]. A handful of initial studies in humans have revealed associations between childhood obesity and DNA methylation [10]. These associations are the results of approaches from two main perspectives. While some authors have demonstrated significant associations between childhood or adolescent obesity and DNA methylation in peripheral blood [11,12,13], others have provided evidence that placenta and umbilical cord (blood and/or tissue) methylation might influence anthropometric characteristics at birth [14,15,16,17] and body composition later in life [18,19,20]. Some of the above-mentioned studies focused on targeted genomic regions with a known relationship with obesity (i.e., LEP/LEPR, ADIPOQA/ADIPOR, PPARG, IGF2, and others) [10], while others, performed epigenome-wide scale analyses that shed light on new genes that were mainly implicated in metabolism and immune regulation [20,21,22].

Placenta and umbilical cord tissues are of extra-embryonic mesoderm origin and give support to fetal development [23]. Both tissues are affected by in utero conditions, and developmental abnormalities in these tissues may cause adverse events in the mother and the child [24,25]. During the last decade, there has been a great interest in the use of these tissues as raw materials for medical applications because of their exceptional properties and the ease of access to them [26]. The majority of clinical studies have utilized placental tissues and umbilical cord veins; however, extensive preclinical experimentation has been performed on the umbilical cord tissue (including Wharton’s Jelly) [27].

Given the above-mentioned findings, we hypothesize that epigenetic signatures established during development can be influenced by maternal obesity and have an effect on the offspring phenotype. Epigenetic marks shared by different tissues may be the most likely related to offspring phenotype. The early detection of such signatures in fetal tissues at birth may also provide predictive markers for the subsequent phenotype of the offspring. Thus, we have used an epigenomic approach to study the impact of maternal pregestational BMI on the offspring’s methylome in paired placenta and umbilical cord tissue samples and the potential effect on their metabolic phenotype in late life.

## 2. Materials and Methods

### 2.1. Study Population and Ethics

The study population consisted of a population-based prenatal cohort of pregnant women who were recruited during pregnancy (in Girona, northeastern Spain) and whose infants were followed-up at 6 years old. The inclusion criteria were: Caucasian origin, infants born at term (37 to 40 weeks), singleton pregnancies, and informed written consent. The exclusion criteria were as follows: women with major medical, surgical, or obstetrical complications, including gestational diabetes, hypertension, or preeclampsia; fetal growth restriction; newborn malformations or asphyxia; assisted reproductive technology; and alcohol abuse or drug addiction during pregnancy.

A total of 114 mother–infant pairs were included in the cohort, of which, 24 were selected for the study of the genome-wide DNA methylation array and 90 for the validation of the top differentially methylated CpGs and gene expression (Supplementary Figure S1). The sampling method was designed to ensure that the sample is representative of the study subjects taking into account pregestational BMI, age, and newborn’s gender.

### 2.2. Mother–Newborn Assessments

Information on pregnancy, labor, and delivery characteristics was retrieved from standardized medical records. Gestational age was determined by utilizing the last menstrual period, and whenever feasible, it was verified through ultrasound assessment. Maternal pregestational weight was acquired through a questionnaire and cross-verified using clinical records. Pregestational BMI was calculated as weight divided by height squared (Kg/m^2^).

Newborns were weighed and measured immediately after delivery using a calibrated scale and a measuring board, respectively. Gestational age- and sex-adjusted z-scores (SDS) for birth weight and length were calculated using regional norms [28].

### 2.3. Umbilical Cord and Placenta Samples

Upon delivery, the umbilical cord was clamped and cut, and a section of the cord (a piece measuring 1 to 4 cm in length) was immediately stored at −80 °C. For the placenta, three cuboidal biopsies (1 cm^3^) from randomly selected lobes of the inner surface of the placenta (maternal side) were obtained. Afterward, the decidua (outermost layer) was removed, samples were washed with a physiological saline buffer to remove the maternal blood, and samples were immediately stored at −80 °C.

### 2.4. Children Assessments

Children whose parents agreed to participate in the follow-up study at 6 years of age (*n* = 62) were examined in the morning under a fasting state, and a venous blood sample was obtained. Their characteristics at birth did not differ from those who did not participate in the follow-up study.

Weight was measured on a calibrated scale, wearing light clothes, and height was measured with a Harpenden stadiometer without shoes. BMI and age- and sex-adjusted z-scores were calculated as above. Waist circumference was measured in the supine position at the umbilical level. The changes between weight at birth and BMI at 6 years (∆ BW-BMI SDS) were calculated as the subtraction between BMI SDS at 6 years and birth weight (BW) SDS. Fat mass (FM) was assessed by bioelectric impedance (Hydra Bioimpedance Analyzer 4200; XiTRON Technologies, San Diego, CA, USA) and it was calculated as body weight minus lean mass. An electronic oscillometer (Dinamap^®^Pro 100; GE Medical Systems, Chalfont St. Giles, UK) with appropriate cuff size was used to measure blood pressure. It was measured on the right arm, after a 10-min rest, and the patient was in a supine position. Data are presented as the average of two measurements. Perirenal fat was measured by a high-sensitivity ultrasound using a convex 3–3.5 MHz transducer placed parallel to the surface of the kidney. Longitudinal scans were performed and the thickness from the inner side of the abdominal musculature to the surface of the kidney was measured.

Serum glucose was measured by the hexokinase method as mg/dL. Insulin was measured by immunochemiluminescence (IMMULITE 2000, Diagnostic Products, Los Angeles, CA, USA) as mIU/L. The lower detection limit was 0.4 mIU/L, and the intra- and inter-assay coefficients of variation (CVs) were <10%. Insulin resistance was estimated from fasting insulin and glucose levels using the homeostasis model assessment [HOMA-IR = (fasting insulin in mIU/L) × (fasting glucose in mM)/22.5]. Total serum triacylglycerol (TG) was measured by monitoring the reaction of glycerol–phosphate oxidase (ARCHITECT, Abbott Laboratories, Abbott Park, IL, USA); the lower detection limit was 5.0 mg/dL and intra- and inter-assay CVs were <5%. High-density lipoprotein cholesterol (HDL-cholesterol) was quantified by a homogenous method of selective detergent with accelerator (ARCHITECT, Abbott Laboratories, Abbott Park, IL, USA); the lower detection limit was 2.5 mg/dL and intra- and inter-assay CVs were <4%.

### 2.5. Genome-Wide DNA Methylation Array

DNA methylation profiling was performed in 24 paired placenta and umbilical cord samples using the Infinium^®^ MethylationEPIC BeadChip array (Illumina, San Diego, CA, USA), which examines >850,000 CpGs across the genome. The process was conducted at the Epigenomics Unit and Biostatistics Service from IIs La Fe (Valencia, Spain) following the manufacturer’s protocol, as previously described [29]. Raw IDAT files were normalized using functional normalization with R package minfi (version 1.28.0). Every beta value in the EPIC array was accompanied by a detection *p*-value, which represents the confidence of a given beta value. Filters were applied as follows: CpGs with a detection *p* value > 0.01 were removed from the analysis, and CpGs associated with SNPs and CpGs located in sexual chromosomes were also removed before the analysis of the data. After the filtering, the remaining 841,818 CpGs were considered valid for the analysis.

The differentially methylated CpGs associated with maternal pregestational BMI were identified using Beta regression models with pregestational BMI as a predictor and the methylation level of each CpG as a response. To correct for multiple comparisons, *p* values were subsequently adjusted using the false discovery rate. All statistical analyses were performed using R (version 3.5.1, RStudio Inc, Vienna, Austria) and R package betareg (version 2.0-13, RStudio Inc, Vienna, Austria). Adjusted *p*-values < 0.05 were considered statistically significant. Given that pregestational BMI is a continuous variable whose range in the sample spans from 18.9 to 38.0, an odds ratio (OR) value higher than 1.05 or lower than 0.95 was considered to have a more relevant biological effect.

Each CpG on the array was assigned to functional regions of the gene, including promoter regions (TSS1500, TSS200, and 5′-UTR), first exon, gene body, and 3′UTR, as well as localization in relation to CpG islands (shore, island, and shelf). Methylation array data were deposited in the Gene Expression Omnibus database (accession number GSE192812).

The WebGestalt (web-based gene set analysis toolkit) was used to identify the metabolic pathways epigenetically affected by maternal pregestational BMI; bioinformatic analyses with DAVID (Database for Annotation, Visualization, and Integrated Discovery) [30,31] were conducted to study the potentially affected diseases and disorders.

### 2.6. CpG Methylation by Pyrosequencing

The top differentially methylated CpGs shared between the placenta and umbilical cord (annotating for *HADHA* and *SLC2A8* genes) were selected for validation in 90 paired placenta and umbilical cord samples by pyrosequencing bisulfite-treated DNA. Genomic DNA was isolated from placenta and umbilical cord samples using the QIAamp DNA mini kit (Qiagen, Hilden, Germany). Sodium bisulfite conversion of 500 ng of DNA was performed using the EpiTect Fast DNA Bisulfite Kit (Qiagen). Bisulfite-treated DNA (10 ng) was PCR-amplified with 2 μM of forward and biotinylated reverse primers (Supplementary Table S1). Both PCR and sequencing primers were designed with the usage of PyroMark Assay Design 2.0 software (Qiagen). The PCR product was rendered single-stranded through biotin capture on magnetic beads and then annealed to the sequencing primer (4 mM) to be subsequently pyro-sequenced in PyroMark Q48 Instrument (Qiagen). CpG site methylation was quantified with the PyroMark Q48 (Qiagen). Raw data were analyzed using the PyroMark Q48 AutoPREP Software V2.4.2 (Qiagen) and the percentage of methylation for each analyzed CpG was obtained.

### 2.7. Gene Expression by Real-Time PCR

The expression levels of the two commonly differentially methylated genes in both placenta and umbilical cord (HADHA and SLC2A8 genes) were studied in 90 paired placenta and umbilical cord samples by RT-qPCR. Total RNA was isolated and retro-transcribed using the RNeasy mini kit (Qiagen, Germany) and the High-Capacity cDNA Reverse Transcription Kit (Applied Biosystems, Waltham, MA, USA). TaqMan Gene Expression assays (Thermo Fisher Scientific, Waltham, MA, USA) were used to amplify the HADHA (Hs00426191_m1) and SLC2A8 (Hs00205863_m1) genes in both placenta and umbilical cord, the housekeeping genes TBP (Hs00427620_m1) and SDHA (Hs00188166_m1) in placenta, and GAPDH (Hs02786624_g1) in umbilical cord. Reactions were run on a LightCycler 480 Real-Time PCR System (Roche Diagnostics, Rotkreuz, Switzerland), using the default cycling conditions. Relative gene expression levels were calculated according to the 2^−ΔCt^ method.

### 2.8. Statistical Methods

Statistical analyses were performed using SPSS Statistics 22.0 (SPSS, Chicago, IL, USA). Results are shown as mean ± standard error of the mean (SEM). An unpaired *t* test was used to study differences between quantitative variables and the chi-square test was used for categorical variables. To study the relationship between epigenetics and offspring parameters, subjects were grouped according to their methylation and expression levels as follows: low levels (those with methylation/expression levels below the 50th percentile of the sample, *n* = 31) and high levels (those with methylation/expression levels above the 50th percentile of the sample, *n* = 31). The univariate general linear model adjusting for potential confounders (maternal pregestational BMI, gestational age, and child’s sex and age) was performed. Statistical significance was set at *p*-value ≤ 0.05.

## 3. Results

### 3.1. Maternal and Offspring Characteristics

Table 1 summarizes the maternal and offspring characteristics of the study population (24 pregnant women included for the genome-wide DNA methylation array and 90 pregnant women included for the validation of the top differentially methylated CpGs and gene expressions). Both groups had similar characteristics, but the validation group displayed slightly lower gestational age and birth weight, and the offspring at 6 years were slightly younger and shorter (all *p* < 0.05).

### 3.2. Genome-Wide DNA Methylation Array

**Figure 1:**
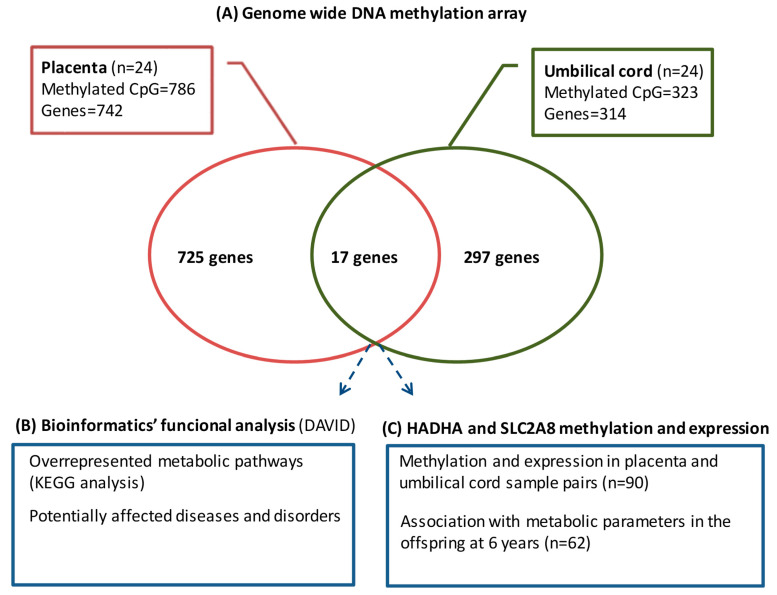
Overview of the analytical strategy used to identify placental and umbilical cord methylated genes, showing the association with pregestational BMI.

The genome-wide DNA methylation array showed that pregestational BMI was associated with differential DNA methylation at 1031 CpGs in placenta (786 CpGs with Ref Seq-annotated genes) and 369 CpGs in umbilical cord (323 CpGs with Ref Seq-annotated genes). We considered those CpGs with Ref Seq-annotated genes to have a greater probability of eliciting significant changes in gene expression and, thus, focused our analyses only on those CpGs with gene annotation. Those CpGs were distributed over 742 and 314 different genes in placenta and umbilical cord, respectively. A subset of 17 genes was commonly differentially methylated in both placenta and umbilical cord (Figure 1A). This indicates that about 3% (in placenta) and 6% (in umbilical cord) of the methylome was shared between both tissues.

**Figure 2:**
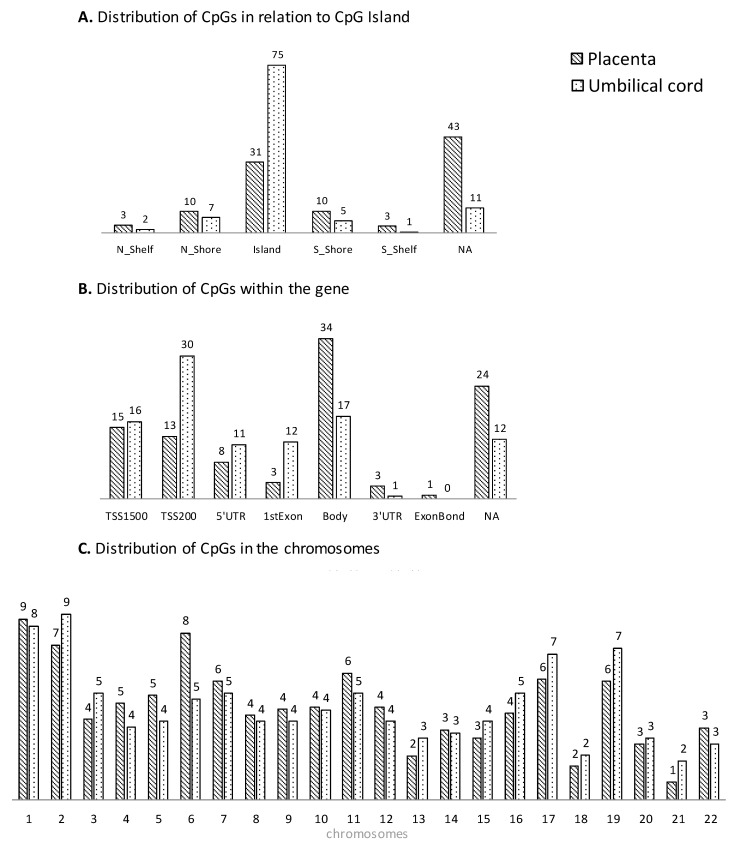
Genomic distribution (% CpG sites) associated with pregestational BMI in placenta (stripped plots) and umbilical cord (dotted plots) in relation to CpG Island (**A**), within the gene (**B**) and the chromosomes (**C**).

The distribution of gene regulatory regions for CpGs with significant changes in DNA methylation is presented in Figure 2A,B. In placenta, the methylated CpGs associated with maternal pregestational BMI were mainly located in a CpG Island (31.4%) and within the gene body (34%) according to the gene region; in umbilical cord, they were mainly located in a CpG Island (74.5%) and within the TSS200 site (30%), according to the gene region. Regarding the distribution within the gene, an excess of CpGs can be observed in all regions before the body (TSS1500, TSS200, 5′UTR, and first exon) in umbilical cord compared to placenta. Additionally, CpGs were widely spread across different chromosomes, indicating that both the placenta and umbilical cord tissue have extensive and significant methylation changes in a genome-wide manner (Figure 2C).

A summary of the top differentially methylated CpGs with the most relevant association with pregestational BMI in placenta and umbilical cord (OR > 1.05 and OR < 0.95) is presented in Table 2 (the full list of differentially methylated CpGs associated with pregestational BMI is shown in Supplementary Table S2). The CpGs that were annotated to *HADHA* and *SLC2A8* genes were placed among the top differentially methylated CpGs in both placenta and umbilical cord.

### 3.3. Bioinformatics Functional Analysis

To establish whether the differentially methylated CpGs had significant functional roles, we conducted bioinformatics analyses, aimed to determine the gene metabolic pathways overrepresented by all the differentially methylated CpGs in placenta and umbilical cord (KEGG analysis) and the potentially related disease/disorders (Figure 1B).

The KEEG analysis of the differentially methylated CpGs in placenta and umbilical cord is shown in Table 3. Differential methylation in placenta showed enrichment in pathways with potentially important effects on extracellular matrix interaction, fatty acid elongation, RNA transport, the AGE-RAGE signaling pathway in diabetic complications, and arrhythmogenic right ventricular cardiomyopathy (ARVC). In umbilical cord, enrichment was observed in pathways related to natural killer cell-mediated cytotoxicity, NOD-like receptor signaling, and RNA transport.

Table 4 shows the top-ranked common diseases/disorders related to the differentially methylated genes in placenta and umbilical cord. The differentially methylated genes in placenta were mainly related to psychological, chemo-dependency, pharmacogenomic, and metabolic diseases. A higher number of genes were involved in tobacco, type 2 diabetes, and renal failure. The differentially methylated genes in umbilical cord were mainly related to pharmacogenomic, developmental, immune, and metabolic diseases. A higher number of genes were involved in type 2 diabetes, cleft lip, and obesity.

### 3.4. HADHA and SLC2A8 Methylation and Expression

Given that HADHA and SLC2A8 were placed among the top methylated CpGs in both placenta and umbilical cord, they were selected to be validated in a wider population by pyrosequencing and RT-qPCR (Figure 1C). The levels of CpG methylation and gene expression for both tissues are compiled in Supplementary Table S3.

**Figure 3:**
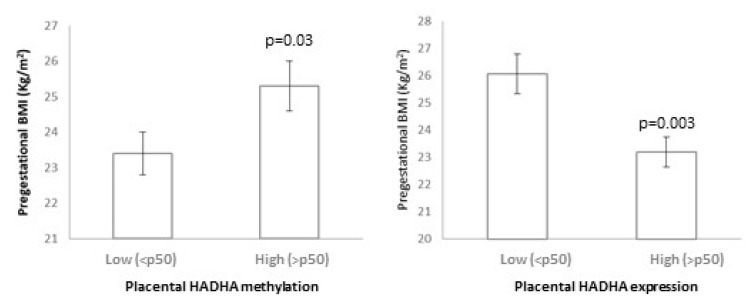
Relationship between placental *HADHA* methylation and expression with pregestational BMI.

Placental HADHA methylation showed a positive relation with pregestational BMI, as mothers with higher placental HADHA methylation (>p50) showed higher pregestational BMI (*p* = 0.03), and placental HADHA expression showed a negative relation with pregestational BMI, as mothers with higher placental HADHA expression (>p50) showed lower pregestational BMI (*p* = 0.003) (Figure 3). In turn, the placental HADHA expression was negatively associated with the methylation levels (*p* = 0.04). Moreover, lower placental HADHA methylation and higher HADHA expression were related to a worse metabolic profile in the offspring at 6 years (higher weight SDS, BMI SDS, Δ BW-BMI SDS, waist, SBP, insulin, HOMA-IR, and FM SDS; all *p* < 0.05) (Table 5(A)). Most of these results remained significant after adjusting for potential confounding factors. No significant relationship was observed between umbilical cord methylation and/or expression and the offspring outcomes; however, children with lower umbilical cord HADHA methylation tended to show higher BMI-SDS, Δ BW-BMI SDS, and SBP (*p* between 0.05 and 0.08) (Table 5(B)).

Pyrosequencing analysis failed to demonstrate a relationship between SLC2A8 methylation and pregestational BMI. However, lower SLC2A8 methylation and higher SLC2A8 expression in placenta and umbilical cord were both related to a worse metabolic profile in the offspring at 6 years. Specifically, lower levels of placental SLC2A8 methylation were related to higher weight SDS, height SDS, and perirenal fat; and higher levels of placental SLC2A8 expression were related to higher FM-SDS (all *p* < 0.05; Table 6A). Concerning the umbilical cord, lower SLC2A8 methylation was related to higher weight SDS, height SDS, BMI SDS, and perirenal fat; and higher SLC2A8 expression was related to higher TG, glucose, and FM SDS (all *p* < 0.05; Table 6B). Some of these results remained significant after adjusting for potential confounding factors.

## 4. Discussion

In this study, we report, for the first time, the DNA methylation signatures associated with maternal pregestational BMI in paired placenta and umbilical cord tissue samples. The key findings from our study include (1) an in silico approach, showing that those differentially methylated CpGs/genes were likely involved in several metabolic pathways related to cell proliferation and development, with potential effects on metabolic diseases; and (2) the identification of *HADHA* and *SLC2A8* genes, which were differentially methylated in both placenta and umbilical cord tissue and related to several metabolic parameters in the offspring at 6 years.

Our data showed that under obesogenic conditions, such as pregestational obesity, placenta and umbilical cord tissue undergo extensive methylation changes in genomic regions involved in important regulatory pathways. These methylation changes may be relevant to disease processes, including type 2 diabetes and obesity. In this sense, the bioinformatics functional analysis showed enrichment in several pathways related to fatty acids, diabetes, and cardiovascular disease in placenta, and pathways related to the immune response in umbilical cord.

The current evidence suggests that pregestational and gestational maternal BMI are associated with some epigenetic signatures in the mother and the offspring [32,33,34], indicating that some of the effects proposed by the DOHaD (Developmental Origins of Health and Disease) hypothesis may indeed be mediated by epigenetic signatures. However, the high variability of methods applied, together with the multitude of different target tissues analyzed and the small sample sets, have not permitted reaching causative conclusions so far. A recent systematic review [35] identified 31 studies exploring the association between pregestational BMI and DNA methylation profile in maternal tissues (2 in maternal blood and 1 in adipose tissue), placenta (7 in placental tissue) and umbilical cord (21 in cord blood and 3 in cord tissue). Most of them studied single samples and targeted specific CpGs, and none of them studied the methylation of the *HADHA* and *SLC2A8* genes.

The overlap of genes and regulatory pathways between placenta and umbilical cord tissue highlights the potential effects of pregestational obesity on the fetus. Several authors have previously shown that in utero conditions, such as gestational diabetes, preeclampsia and obesity could affect the developing fetus by inducing epigenetic changes in fetal tissues (cord blood and placental) [36,37,38,39], and that differentially methylated regions significantly overlapped between both tissues. However, all the previous works investigated cord blood instead of umbilical cord tissue. Our results showed that placenta and umbilical cord tissue shared about 3–6% of the methylome, being HADHA and SLC2A8 among the top differentially methylated CpGs in both tissues.

The HADHA gene (hydroxyl acyl-CoA dehydrogenase trifunctional multienzyme complex subunit alpha) encodes the alpha subunit of the mitochondrial trifunctional protein, an enzyme that catalyzes the last three steps of fatty acid beta-oxidation [40]. In mice, the reduction of HADHA hepatic proteins led to an increase in triglyceride levels and, consequently, to a fatty liver [41]. Moreover, deficiency of the *HADHA* gene causes fetal growth retardation and neonatal hypoglycemia [42] and those mice heterozygotes for the HADHA allele develop hepatic steatosis and insulin resistance [43]. Non-alcoholic steatohepatitis in rats fed with a high-fat diet resulted in the downregulation of HADHA protein expression in liver cells [44]. In this sense, our results show that pregestational BMI relates to higher methylation and a lower expression of HADHA in placenta. Indeed, the epigenetic changes induced by pregestational BMI may have an impact on the offspring as lower placental HADHA methylation and higher HADHA expression were related to a worse metabolic profile in the offspring at age 6 years (including an increased change in BMI from birth and higher fat mass accumulation and HOMA-IR). We have not come across any studies investigating DNA methylation in the HADHA gene; only genome-wide DNA methylation in end-stage human heart failure showed that differential promoter methylation of several metabolic intermediates (including HADHA) in heart tissue was associated with the expression of the same genes in opposite direction [45]. In agreement with these results, our studied HADHA CpGs were associated with inverse gene expression.

The *SLC2A8* gene (Solute carrier family 2 member 8), also known as GLUT8, is a glucose and fructose transporter expressed in highly oxidative tissues that regulates metabolic homeostasis [46]. GLUT8 deficiency prevents fructose-induced fat accumulation, glycemic dysregulation, and dyslipidemia through PPARγ and its downstream targets [47]. SLC2A8 is expressed by the human placental trophoblast during pregnancy [48] and has previously been related to reduced fetal growth [49]; SLC2A8 was hypomethylated in the peripheral blood of small-for-gestational-age (SGA) infants aged 12 months and negatively associated with obesity measures (BMI z-scores and fat mass) at 12 and 24 months [50]. Similarly, our results showed that pregestational BMI relates to SLC2A8 hypomethylation in both placenta and umbilical cord in the methylation array. Children with higher SLC2A8 methylation showed decreased weight, height, BMI, and perirenal fat accumulation. Thus, SLC2A8 methylation could play a role in the prevention of the obesity-related phenotype induced by pregestational obesity. We acknowledge that our validation analysis failed to demonstrate a relationship between SLC2A8 methylation and pregestational BMI; this could be due to technical issues (the microarray technology is different from the pyrosequencing technology) or the characteristics of the study population, which could differ in other parameters not studied in this work (i.e., medication, diet, and exercise).

Our data suggest that methylation changes induced by pregestational obesity can be similar in placenta and umbilical cord tissue. This may indicate that multiple other tissues and cells experience similar fetal epigenetic programming, due to the same in utero metabolic environmental exposure, and may be part of the mechanisms leading to long-term offspring adverse outcomes. However, further work is needed to determine the relevance of epigenetic changes in metabolic fetal programming. In fact, several differences have been observed in the CpG distribution between placenta and umbilical cord, mainly regarding the distribution within the gene. Recent data show that for some genomic regions, methylation appears largely independent of the tissue of origin, whereas for others, there is a clear tissue-specific dependence [51].

We acknowledge that our study has some limitations. One factor worth considering is the heterogeneity of cells present in the studied tissues. Recent data suggest that the variation in cell-type proportions across samples may confound associations of DNA methylation with modeled outcomes; cell-type deconvolution approaches are being developed to infer cell-type proportions and give insights into cell-specific methylation effects [52]. In our study, although we did not adjust for cell-type proportions, the tissue samples were fully homogenized to ensure homogeneity of the sample and ease the potential implementation at a clinical level without previous cell-sorting requirements. In this study, the comparison of DNA methylation signatures between placenta and umbilical cord samples allows us to identify only a single CpG. The identification of several CpGs from a differentially methylated region would have been more relevant and have had a higher biological effect. Another weakness of the study is the lack of paternal information as parental size could be an important confounding factor. We acknowledge that all these limitations may affect our results and may have precluded us from identifying other interesting genes and/or reaching relevant conclusions.

Although our data compare children with higher and lower levels of methylation and gene expression and, thus, can only imply a relationship between DNA methylation at birth and the later phenotype, the importance of the observation stands, irrespective of whether the methylation is causally related to the increased risk for obesity. Despite being merely a non-causal association, the changed epigenetic status provides a potential marker of the altered developmental trajectory by the time of birth, which may have prognostic value and potential utility for monitoring programs to optimize maternal health and nutrition to provide long-term benefits to the offspring.

The epigenetic marks identified in this study could be used (1) as novel biomarkers that may be determined in clinical laboratories at birth to diagnose the susceptibility of the offspring to developing overweight/obesity and associated metabolic diseases; (2) to develop therapeutic strategies aimed at reversing the occurrence of epigenetic changes and preventing subsequent metabolic implications.

## 5. Conclusions

In conclusion, by using DNA methylation signatures related to maternal pregestational BMI from two different neonatal tissues (placenta and umbilical cord tissue), we provide the epigenetic status of a broader spectrum of genes involved in several metabolic pathways related to cell proliferation and development, with potential effects on metabolic diseases. Moreover, we provide evidence that the methylation of the HADHA and SLC2A8 genes is associated with several metabolic parameters in the offspring at 6 years. This suggests that the epigenetic modifications induced by pregestational obesity may be of functional relevance in the offspring’s metabolic phenotype.

## Figures and Tables

**Table 1:** Clinical characteristics of the study subjects.

	Array	Validation	*p* Value
Mother (n)	24	90	
Age (yr)	31 ± 1	31 ± 1	Ns
Height (cm)	164 ± 1	163 ± 1	Ns
Pregestational weight (kg)	68.5 ± 2.9	65.3 ± 1.4	Ns
Pregestational BMI (Kg/m^2^)	25.2 ± 1.0	24.5 ± 0.4	Ns
Pregestational Obesity (%)	31	33	Ns
Pre-delivery weight (kg)	84.7 ± 2.9	79.6 ± 1.4	Ns
Pre-delivery BMI (Kg/m^2^)	31.2 ± 1.1	29.9 ± 0.5	Ns
Gestational weight gain (Kg)	16.1 ± 0.6	14.3 ± 0.5	Ns
Newborn (n)	24	90	
Gender (% female)	50	43	Ns
Gestational age (wk)	40 ± 0.1	39 ± 0.1	0.04
Birth weight (Kg)	3.4 ± 0.1	3.2 ± 0.1	0.01
Birth weight SDS	0.30 ± 0.1	0.01 ± 0.1	0.04
Birth length(cm)	50.1 ± 0.2	49.5 ± 0.1	Ns
Birth length SDS	0.07 ± 0.1	−0.21 ± 0.1	Ns
Offspring at 6 yr (n)	24	62	
Gender (% female)	50	43	Ns
Age (yr)	6.2 ± 0.1	5.8 ± 0.1	0.04
Weight (kg)	23.7 ± 1.0	22.3 ± 0.6	Ns
Weight SDS	0.22 ± 0.2	0.13 ± 0.1	Ns
Height (cm)	120 ± 1	114 ± 1	0.004
Height SDS	0.46 ± 0.2	−0.07 ± 0.1	Ns
BMI (kg/m^2^)	16.3 ± 0.3	16.8 ± 0.3	Ns
BMI SDS	−0.02 ± 0.1	0.24 ± 0.1	Ns
∆ BW-BMI SDS	−0.30 ± 0.2	0.23 ± 0.1	Ns
Waist (cm)	57.1 ± 1.7	57.1 ± 0.9	Ns
Hip (cm)	61.1 ± 1.8	59.9 ± 1.0	Ns
SBP (mmHg)	96.9 ± 3.0	96.2 ± 1.4	Ns
DBP (mmHg)	57.1 ± 1.1	55.9 ± 0.9	Ns
HDL-cholesterol (mg/dL)	57.0 ± 2.7	54.4 ± 1.2	Ns
Triglycerides (mg/dL)	49.5 ± 2.7	50.8 ± 2.1	Ns
Glucose (mg/dL)	85.0 ± 1.7	82.5 ± 1.0	Ns
Insulin (mIU/L)	6.2 ± 0.5	5.5 ± 0.2	Ns
HOMA-IR	1.3 ± 0.1	1.1 ± 0.1	Ns
Perirenal fat (cm)	0.10 ± 0.1	0.11 ± 0.1	Ns
FM (Kg)	5.9 ± 0.6	5.7 ± 0.4	Ns
FM SDS	0.37 ± 0.3	0.59 ± 0.2	Ns

Data are shown as mean ± SEM. BMI: body mass index; SDS: standard deviation score; ∆ BW-BMI SDS: z score changes from weight at birth to BMI at 6 years; SBP: systolic blood pressure; DBP: diastolic blood pressure; HDL: high-density lipoprotein; HOMA-IR: Homeostatic model assessment for insulin resistance; FM: fat mass; Ns: non-significant.

**Table 2:** Summary of the top differentially methylated CpGs (selected by odds ratio) and their annotated genes in placenta (**A**) and umbilical cord (**B**).

**(A) Placenta**						
**IlmnID**	**Beta Coefficient**	**FDR-Adjusted *p*-Value**	**OR**	**Chr**	**Position**	**Gene Name**
**cg13692482**	−0.130	2.04 × 10^−8^	0.878	9	130168651	***SLC2A8***
cg13031029	−0.124	3.34 × 10^−6^	0.883	7	107574783	*LAMB1*
cg03357803	−0.106	3.76 × 10^−5^	0.899	15	28272345	*OCA2*
cg12006733	−0.105	2.17 × 10^−5^	0.901	15	90177014	*KIF7*
cg12680750	−0.104	7.92 × 10^−7^	0.901	2	133426182	*LYPD1*
cg04899492	0.096	5.82 × 10^−5^	1.101	6	24126312	*NRSN1*
cg05888872	0.098	1.23 × 10^−7^	1.103	10	14050479	*FRMD4A*
**cg01188578**	0.103	2.48 × 10^−5^	1.108	2	26464058	***HADHA***
cg16936887	0.105	5.28 × 10^−5^	1.111	18	52989025	*TCF4*
cg12877278	0.117	3.59 × 10^−7^	1.124	6	151551059	*LOC102723831*
**(B) Umbilical Cord**						
**IlmnID**	**Beta Coefficient**	**FDR-Adjusted *p*-Value**	**OR**	**Chr**	**Position**	**Gene Name**
**cg13692482**	−0.133	2.35 × 10^−6^	0.875	9	130168651	***SLC2A8***
cg03751055	−0.084	7.46 × 10^−6^	0.919	10	131380455	*MGMT*
cg23143502	−0.063	3.64 × 10^−7^	0.939	4	675936	*MFSD7*
cg25783997	−0.058	7.21 × 10^−6^	0.944	2	185462928	*ZNF804A*
cg16396228	−0.050	3.06 × 10^−6^	0.952	17	46985589	*UBE2Z*
cg03876184	0.074	1.31 × 10^−5^	1.077	2	27886755	*SUPT7L*
cg04740665	0.080	8.39 × 10^−6^	1.083	1	205560997	*MFSD4*
cg16513685	0.081	6.31 × 10^−7^	1.084	9	33738831	*LINC01251*
cg26028074	0.118	1.39 × 10^−6^	1.125	17	32201533	*ASIC2*
**cg01188578**	0.179	2.16 × 10^−6^	1.196	2	26464058	***HADHA***

FDR: false discovery rate; OR: odds ratio; Chr: chromosome. In bold, the CpGs/genes shared between placenta and umbilical cord.

**Table 3:** Enriched Kyoto Encyclopedia of Genes and Genomes (KEEG) pathways of methylated CpGs associated with maternal pregestational BMI in placenta and umbilical cord. The number of genes involved in each pathway and the *p* value are shown.

**Placenta**	**Genes**	***p* Value**
Hsa04512: ECM–receptor interaction	3	0.016
Hsa00062: Fatty acid elongation	4	0.018
Hsa03013: RNA transport	7	0.031
Hsa04933: AGE-RAGE signaling pathway in diabetic complications	4	0.036
Hsa05412: Arrhythmogenic right ventricular cardiomyopathy (ARVC)	3	0.045
**Umbilical Cord**	**Genes**	***p* Value**
Hsa04650: Natural killer cell-mediated cytotoxicity	3	0.007
Hsa04621: NOD-like receptor signaling pathway	7	0.010
Hsa03013: RNA transport	4	0.047

**Table 4:** Top-ranked common diseases and disorders to which differentially methylated genes in placenta and umbilical cord belonged. The number of genes involved in each disease/disorder and the *p* value are shown.

**Placenta**	**Genes**	***p* Value**
Tobacco User Disorder	139	0.0078
Height	17	0.014
Eosinophils	8	0.016
Hemoglobin A, Glycosylated	14	0.016
Coronary Disease	17	0.02
Attention Deficit and Disruptive Behavior Disorders	4	0.021
Bone Mineral Density	25	0.024
Occipital Lobe	4	0.029
Type 2 Diabetes	100	0.032
Intuition	3	0.034
Pancreatic Neoplasms	6	0.049
Breath Tests	10	0.05
Chronic Renal Failure	45	0.05
**Umbilical Cord**	**Genes**	***p* Value**
Type 2 Diabetes	57	0.00009
Cleft Lip	14	0.0092
Obesity	13	0.013
Autism	12	0.025
Hypertension	19	0.028
Respiratory Function Tests	9	0.042
Leukemia	8	0.05

**Table 5:** Relationship between *HADHA* methylation, expression, and offspring parameters at 6 years in (**A**) placenta and (**B**) umbilical cord.

**(A) Placenta**						
	**HADHA Methylation**			**HADHA Expression**		
	**Low Levels (<p50)**	**High Levels (>p50)**	***p*** **Value**	**Low Levels (<p50)**	**High Levels (>p50)**	***p*** **Value**
Weight SDS	0.57 ± 0.25	−0.10 ± 0.15	**0.02**	−0.19 ± 0.17	0.41 ± 0.22	**0.03**
Height SDS	0.03 ± 0.25	−0.14 ± 0.19	Ns	−0.31 ± 0.21	0.13 ± 0.19	Ns
BMI SDS	0.58 ± 0.24	−0.01 ± 0.15	**0.03**	−0.03 ± 0.13	0.56 ± 0.26	**0.03**
∆ BW-BMI SDS	0.67 ± 0.25	−0.13 ± 0.19	**0.01**	−0.13 ± 0.16	0.51 ± 0.29	**0.04**
Waist	58.8 ± 1.4	56.1 ± 1.1	Ns	55.0 ±0.8	58.6 ± 1.6	**0.04**
SBP	101.0 ± 2.1	92.3 ± 1.6	**0.002**	94.3 ± 1.9	98.8 ± 2.1	Ns
DBP	56.9 ± 1.2	55.4 ± 1.4	Ns	55.6 ± 1.3	56.3 ± 1.4	Ns
HDL-cholesterol	56.4 ± 1.8	52.9 ± 1.7	Ns	54.5 ± 1.9	54.5 ± 1.5	Ns
Triglycerides	53.0 ± 3.4	48.8 ± 2.6	Ns	51.0 ± 3.0	50.4 ± 2.9	Ns
Glucose	83.7 ± 1.3	81.2 ± 1.4	Ns	82.0 ± 1.3	82.9 ± 1.5	Ns
Insulin	6.1 ± 0.3	5.0 ± 0.4	**0.04**	4.87 ± 0.32	6.10 ± 0.48	0.03
HOMA-IR	1.28 ± 0.10	1.03 ± 0.09	**0.04**	0.95 ± 0.07	1.21 ± 0.11	0.04
Perirenal fat	0.10 ±0.01	0.11 ± 0.01	Ns	0.11 ± 0.01	0.10 ± 0.01	Ns
FM SDS	1.11 ± 0.39	0.01 ± 0.23	**0.01**	0.02 ± 0.26	1.12 ± 0.39	**0.02**
**(B) Umbilical Cord**						
	**HADHA Methylation**			**HADHA Expression**		
	**Low Levels (<p50)**	**High Levels (>p50)**	***p*** **Value**	**Low Levels (<p50)**	**High Levels (>p50)**	***p*** **Value**
Weight SDS	0.16 ± 0.28	0.03 ± 0.17	Ns	0.49 ± 0.19	−0.09 ± 0.21	0.04
Height SDS	−0.26 ± 0.26	0.04 ± 0.23	Ns	0.27 ± 0.19	−0.41 ± 0.20	0.01
BMI SDS	0.36 ± 0.25	0.03 ± 0.18	Ns	0.29 ± 0.19	0.15 ± 0.19	Ns
∆ BW-BMI SDS	0.34 ± 0.26	−0.07 ± 0.24	Ns	0.15 ± 0.23	0.27 ± 0.21	Ns
Waist	55.6 ± 1.2	56.1 ± 1.2	Ns	57.3 ± 1.1	56.8 ± 1.4	Ns
SBP	97.4 ± 2.4	92.8 ± 2.1	Ns	97.0 ± 1.6	96.0 ± 2.2	Ns
DBP	54.9 ± 1.3	54.2 ± 1.5	Ns	57.8 ± 1.3	54.7 ± 1.3	Ns
HDL-cholesterol	55.85 ± 1.92	53.56 ± 1.97	Ns	53.6 ± 1.6	54.9 ± 1.8	Ns
Triglycerides	46.41 ± 2.43	54.56 ± 3.96	Ns	52.1 ± 3.2	49.3 ± 2.5	Ns
Glucose	82.07 ± 1.53	80.68 ± 1.57	Ns	82.67 ± 1.47	82.36 ± 1.28	Ns
Insulin	5.13 ± 0.39	5.12 ± 0.43	Ns	5.71 ± 0.41	5.36 ± 0.38	Ns
HOMA-IR	1.07 ± 0.09	1.01 ± 0.08	Ns	1.18 ± 0.10	1.11 ± 0.08	Ns
Perirenal fat	0.11 ± 0.01	0.10 ± 0.01	Ns	0.10 ± 0.01	0.11 ± 0.01	Ns
FM SDS	0.51 ± 0.39	0.28 ± 0.33	Ns	0.61 ± 0.34	0.48 ± 0.32	Ns

Data are presented as mean ± standard error of mean (SEM). BMI: body mass index; SBP: systolic blood pressure; DBP: diastolic blood pressure; HDL: high-density lipoprotein; HOMA-IR: homeostatic model assessment of insulin resistance; FM: fat mass; Ns: non-significant. *p* Value was obtained using *t* test. Significant results after adjustment for confounding variables in univariate general linear models are shown in bold.

**Table 6:** Relationship between *SLC2A8* methylation, expression, and offspring parameters at 6 years in (**A**) placenta and (**B**) umbilical cord.

**(A) Placenta**						
	**SLC2A8 Methylation**			**SLC2A8 Expression**		
	**Low Levels (<p50)**	**High Levels (>p50)**	***p*** **Value**	**Low Levels (<p50)**	**High Levels (>p50)**	***p*** **Value**
Weight SDS	0.80 ± 0.31	0.01 ± 0.18	0.02	0.19 ± 0.21	−0.01 ± 0.19	Ns
Height SDS	0.75 ± 0.19	−0.13 ± 0.20	**0.007**	−0.19 ± 0.18	0.01 ± 0.24	Ns
BMI SDS	0.50 ± 0.31	0.23 ± 0.20	Ns	0.37 ± 0.20	0.01 ± 0.18	Ns
∆ BW-BMI SDS	0.51 ± 0.35	0.18 ± 0.22	Ns	0.47 ± 0.22	−0.13 ± 0.21	Ns
Waist	58.4 ± 2.0	57.6 ± 1.3	Ns	56.0 ± 1.2	57.4 ± 1.3	Ns
SBP	96.0 ± 2.1	95.9 ± 1.8	Ns	96.3 ± 2.3	96.4 ± 1.6	Ns
DBP	56.5 ± 1.7	55.4 ± 1.5	Ns	55.8 ± 1.2	56.1 ± 1.5	Ns
HDL-cholesterol	55.1 ± 3.0	55.9 ± 1.4	Ns	52.9 ± 1.7	56.2 ± 1.8	Ns
Triglycerides	47.8 ± 3.5	50.7 ± 2.7	Ns	50.8 ± 3.1	50.7 ± 2.8	Ns
Glucose	80.9 ± 1.5	82.8 ± 1.6	Ns	81.8 ± 1.2	83.1 ± 1.6	Ns
Insulin	5.6 ± 0.4	5.5 ± 0.4	Ns	5.3 ± 0.4	5.6 ± 0.3	Ns
HOMA-IR	1.14 ± 0.1	1.17 ± 0.1	Ns	1.10 ± 0.09	1.17 ± 0.10	Ns
Perirenal fat	0.13 ± 0.01	0.10 ± 0.01	**0.02**	0.12 ± 0.01	0.10 ± 0.01	Ns
FM SDS	1.02 ± 0.47	0.74 ± 0.36	Ns	−0.15 ± 0.17	0.78 ± 0.36	0.02
**(B) Umbilical Cord**						
	**SLC2A8 Methylation**			**SLC2A8 Expression**		
	**Low Levels (<p50)**	**High Levels (>p50)**	***p*** **Value**	**Low Levels (<p50)**	**High Levels (>p50)**	***p*** **Value**
Weight SDS	0.75 ± 0.28	0.01 ± 0.18	0.03	0.05 ± 0.19	0.20 ± 0.21	Ns
Height SDS	0.50 ± 0.23	−0.16 ± 0.18	0.02	0.04 ± 0.21	−0.17 ± 0.20	Ns
BMI SDS	0.67 ± 0.27	0.10 ± 0.18	0.04	0.07 ± 0.16	0.37 ± 0.22	Ns
∆ BW-BMI SDS	0.59 ± 0.32	0.14 ± 0.23	Ns	0.04 ± 0.20	0.38 ± 0.23	Ns
Waist	58.3 ± 1.9	57.7 ± 1.4	Ns	56.0 ± 1.2	58.0 ± 1.2	Ns
SBP	95.0 ± 2.0	96.3 ± 2.0	Ns	94.4 ± 1.8	98.6 ± 2.12	Ns
DBP	55.6 ± 1.9	56.1 ± 1.4	Ns	57.0 ± 1.4	55.5 ± 1.2	Ns
HDL-cholesterol	56.9 ± 2.7	54.7 ± 1.5	Ns	53.9 ± 1.8	54.7 ± 1.7	Ns
Triglycerides	45.6 ± 3.4	51.6 ± 2.8	Ns	45.7 ± 2.3	55.3 ± 3.1	0.02
Glucose	80.0 ± 1.4	83.6 ± 1.6	Ns	80.72 ± 1.30	84.21 ± 1.38	0.05
Insulin	5.2 ± 0.4	5.7 ± 0.4	Ns	5.32 ± 0.41	5.73 ± 0.38	Ns
HOMA-IR	1.0 ± 0.1	1.2 ± 0.1	Ns	1.08 ± 0.09	1.21 ± 0.09	Ns
Perirenal fat	0.13 ± 0.01	0.10 ± 0.01	**0.006**	0.10 ± 0.01	0.11 ± 0.01	Ns
FM SDS	0.75 ± 0.42	0.84 ± 0.38	Ns	0.10 ± 0.26	1.02 ± 0.37	0.04

Data are presented as mean ± standard error of mean (SEM). BMI: body mass index, SDS: standard deviation score; ∆ BW-BMI SDS: z score changes from weight at birth to BMI at 6 years; SBP: systolic blood pressure; DBP: diastolic blood pressure; HDL: high-density lipoprotein; HOMA-IR: homeostatic model assessment of insulin resistance; FM: fat mass; Ns: non-significant. *p* value was obtained using *t* test. Significant results after adjustment for confounding variables in univariate general linear models are shown in bold.

## Data Availability

The datasets generated and analyzed during the current study are available from the corresponding author upon reasonable request. Methylation array data were deposited in the Gene Expression Omnibus database (accession number GSE192812).

## Supplementary Materials

The following supporting information can be downloaded at https://www.mdpi.com/article/10.3390/biomedicines12020301/s1, Figure S1: Flow chart of the subjects included for the array and validation; Table S1: Pyrosequencing primers design and PCR conditions; Table S2: Full list of methylated CpGs associated with pregestational BMI; Table S3: Levels of *HADHA* and *SLC2A8* CpGs methylation (by pyrosequencing) and expression (by RT-qPCR) in placenta and umbilical cord.

## References

[1] Smith G.D., Steer C., Leary S., Ness A. (2007). Is there an intrauterine influence on obesity? Evidence from parent child associations in the Avon Longitudinal Study of Parents and Children (ALSPAC). Arch. Dis. Child..

[2] Warner M.J., Ozanne S.E. (2010). Mechanisms involved in the developmental programming of adulthood disease. Biochem. J..

[3] Poston L. (2012). Maternal obesity, gestational weight gain and diet as determinants of offspring long term health. Best Pract. Res. Clin. Endocrinol. Metab..

[4] Gaillard R., Steegers E.A., Duijts L., Felix J.F., Hofman A., Franco O.H., Jaddoe V.W. (2014). Childhood cardiometabolic outcomes of maternal obesity during pregnancy: The Generation R Study. Hypertension.

[5] Cox B., Luyten L.J., Dockx Y., Provost E., Madhloum N., De Boever P., Neven K.Y., Sassi F., Sleurs H., Vrijens K. (2020). Association Between Maternal Prepregnancy Body Mass Index and Anthropometric Parameters, Blood Pressure, and Retinal Microvasculature in Children Age 4 to 6 Years. JAMA Netw. Open.

[6] Tan H.C., Roberts J., Catov J., Krishnamurthy R., Shypailo R., Bacha F. (2015). Mother’s pre-pregnancy BMI is an important determinant of adverse cardiometabolic risk in childhood. Pediatr. Diabetes.

[7] Sharp G.C., Lawlor D.A., Richmond R.C., Fraser A., Simpkin A., Suderman M., Shihab H.A., Lyttleton O., McArdle W., Ring S.M. (2015). Maternal pre-pregnancy BMI and gestational weight gain, offspring DNA methylation and later offspring adiposity: Findings from the Avon Longitudinal Study of Parents and Children. Int. J. Epidemiol..

[8] Portela A., Esteller M. (2010). Epigenetic modifications and human disease. Nat. Biotechnol..

[9] Wahl S., Drong A., Lehne B., Loh M., Scott W.R., Kunze S., Tsai P.-C., Ried J.S., Zhang W., Yang Y. (2017). Epigenome-wide association study of body mass index, and the adverse outcomes of adiposity. Nature.

[10] Lima R.S., de Assis Silva Gomes J., Moreira P.R. (2020). An overview about DNA methylation in childhood obesity: Characteristics of the studies and main findings. J. Cell. Biochem..

[11] Wang X., Zhu H., Snieder H., Su S., Munn D., Harshfield G., Maria B.L., Dong Y., Treiber F., Gutin B. (2010). Obesity related methylation changes in DNA of peripheral blood leukocytes. BMC Med..

[12] Xu X., Su S., Barnes V.A., De Miguel C., Pollock J., Ownby D., Shi H., Zhu H., Snieder H., Wang X. (2013). A genome-wide methylation study on obesity: Differential variability and differential methylation. Epigenetics.

[13] García-Cardona M.C., Huang F., García-Vivas J.M., López-Camarillo C., Navarro B.E.d.R., Olivos E.N., Hong-Chong E., Bolaños-Jiménez F., Marchat L.A. (2014). DNA methylation of leptin and adiponectin promoters in children is reduced by the combined presence of obesity and insulin resistance. Int. J. Obes..

[14] Engel S.M., Joubert B.R., Wu M.C., Olshan A.F., Håberg S.E., Ueland P.M., Nystad W., Nilsen R.M., Vollset S.E., Peddada S.D. (2014). Neonatal genome-wide methylation patterns in relation to birth weight in the Norwegian Mother and Child Cohort. Am. J. Epidemiol..

[15] Michels K.B., Harris H.R., Barault L. (2011). Birthweight, maternal weight trajectories and global DNA methylation of LINE-1 repetitive elements. PLoS ONE.

[16] St-Pierre J., Hivert M.-F., Perron P., Poirier P., Guay S.-P., Brisson D., Bouchard L. (2012). IGF2 DNA methylation is a modulator of newborn’s fetal growth and development. Epigenetics.

[17] Lesseur C., Armstrong D.A., Paquette A.G., Koestler D.C., Padbury J.F., Marsit C.J. (2013). Tissue-specific Leptin promoter DNA methylation is associated with maternal and infant perinatal factors. Mol. Cell. Endocrinol..

[18] Kochmanski J., Goodrich J.M., Peterson K.E., Lumeng J.C., Dolinoy D.C. (2019). Neonatal bloodspot DNA methylation patterns are associated with childhood weight status in the Healthy Families Project. Pediatr. Res..

[19] Martin C.L., Jima D., Sharp G.C., McCullough L.E., Park S.S., Gowdy K.M., Skaar D., Cowley M., Maguire R.L., Fuemmeler B. (2019). Maternal pre-pregnancy obesity, offspring cord blood DNA methylation, and offspring cardiometabolic health in early childhood: An epigenome-wide association study. Epigenetics.

[20] Agha G., Hajj H., Rifas-Shiman S.L., Just A.C., Hivert M.-F., Burris H.H., Lin X., Litonjua A.A., Oken E., DeMeo D.L. (2016). Birth weight-for-gestational age is associated with DNA methylation at birth and in childhood. Clin. Epigenet..

[21] Relton C.L., Groom A., Pourcain B.S., Sayers A.E., Swan D.C., Embleton N.D., Pearce M.S., Ring S.M., Northstone K., Tobias J.H. (2012). DNA methylation patterns in cord blood DNA and body size in childhood. PLoS ONE.

[22] Simpkin A.J., Suderman M., Gaunt T.R., Lyttleton O., McArdle W.L., Ring S.M., Tilling K., Davey Smith G., Relton C.L. (2015). Longitudinal analysis of DNA methylation associated with birth weight and gestational age. Hum. Mol. Genet..

[23] Roy A., Mantay M., Brannan C., Griffiths S. (2022). Placental Tissues as Biomaterials in Regenerative Medicine. Biomed. Res. Int..

[24] Agarwal P., Morriseau T.S., Kereliuk S.M., Doucette C.A., Wicklow B.A., Dolinsky V.W. (2018). Maternal obesity, diabetes during pregnancy and epigenetic mechanisms that influence the developmental origins of cardiometabolic disease in the offspring. Crit. Rev. Clin. Lab. Sci..

[25] Robinson W.P., Price E.M. (2015). The human placental methylome. Cold Spring Harb. Perspect. Med..

[26] Pogozhykh O., Prokopyuk V., Figueiredo C., Pogozhykh D. (2018). Placenta and Placental Derivatives in Regenerative Therapies: Experimental Studies, History, and Prospects. Stem. Cells Int..

[27] Moore M.C., Van De Walle A., Chang J., Juran C., McFetridge P.S. (2017). Human Perinatal-Derived Biomaterials. Adv. Healthc. Mater..

[28] Carrascosa A., Fernandez J.M., Fernandez C., Ferrandez A., Lopez-Siguero J.P., Sanchez E., Sobradillo B., Yeste D. (2008). Spanish growth studies 2008. New anthropometric standards. Endocrinol. Nutr..

[29] Gómez-Vilarrubla A., Mas-Parés B., Carreras-Badosa G., Xargay-Torrent S., Prats-Puig A., Bonmatí-Santané A., de Zegher F., Ibañez L., López-Bermejo A., Bassols J. (2023). Placental epigenetic marks related to gestational weight gain reveal potential genes associated with offspring obesity parameters. Obesity.

[30] Sherman B.T., Hao M., Qiu J., Jiao X., Baseler M.W., Lane H.C., Imamichi T., Chang W. (2022). DAVID: A web server for functional enrichment analysis and functional annotation of gene lists (2021 update). Nucleic Acids Res..

[31] Huang D.W., Sherman B.T., Lempicki R.A. (2009). Systematic and integrative analysis of large gene lists using DAVID bioinformatics resources. Nat. Protoc..

[32] Shrestha D., Ouidir M., Workalemahu T., Zeng X., Tekola-Ayele F. (2020). Placental DNA methylation changes associated with maternal prepregnancy BMI and gestational weight gain. Int. J. Obes..

[33] Mitsuya K., Parker A.N., Liu L., Ruan J., Vissers M.C.M., Myatt L. (2017). Alterations in the placental methylome with maternal obesity and evidence for metabolic regulation. PLoS ONE.

[34] Ghildayal N., Fore R., Lutz S.M., Cardenas A., Perron P., Bouchard L., Hivert M.-F. (2022). Early-pregnancy maternal body mass index is associated with common DNA methylation markers in cord blood and placenta: A paired-tissue epigenome-wide association study. Epigenetics.

[35] Opsahl J.O., Moen G.-H., Qvigstad E., Böttcher Y., Birkeland K.I., Sommer C. (2021). Epigenetic signatures associated with maternal body mass index or gestational weight gain: A systematic review. J. Dev. Orig. Health Dis..

[36] Bozack A.K., Colicino E., Just A.C., Wright R.O., Baccarelli A.A., Wright R.J., Lee A.G. (2022). Associations between infant sex and DNA methylation across umbilical cord blood, artery, and placenta samples. Epigenetics.

[37] Nomura Y., Lambertini L., Rialdi A., Lee M., Mystal E.Y., Grabie M., Manaster I., Huynh N., Finik J., Davey M. (2014). Global methylation in the placenta and umbilical cord blood from pregnancies with maternal gestational diabetes, preeclampsia, and obesity. Reprod. Sci..

[38] Su R., Wang C., Feng H., Lin L., Liu X., Wei Y., Yang H. (2016). Alteration in Expression and Methylation of IGF2/H19 in Placenta and Umbilical Cord Blood Are Associated with Macrosomia Exposed to Intrauterine Hyperglycemia. PLoS ONE.

[39] Lu S., Wang J., Kakongoma N., Hua W., Xu J., Wang Y., He S., Gu H., Shi J., Hu W. (2022). DNA methylation and expression profiles of placenta and umbilical cord blood reveal the characteristics of gestational diabetes mellitus patients and offspring. Clin. Epigenet..

[40] Rector R.S., Payne R.M., Ibdah J.A. (2008). Mitochondrial trifunctional protein defects: Clinical implications and therapeutic approaches. Adv. Drug Deliv. Rev..

[41] Liao C.C., Lin Y.L., Kuo C.F. (2015). Effect of high-fat diet on hepatic proteomics of hamsters. J. Agric. Food Chem..

[42] Liao C.-C., Lin Y.-L., Kuo C.-F. (2001). Lack of mitochondrial trifunctional protein in mice causes neonatal hypoglycemia and sudden death. J. Clin. Investig..

[43] Ibdah J.A., Perlegas P., Zhao Y., Angdisen J., Borgerink H., Shadoan M.K., Wagner J.D., Matern D., Rinaldo P., Cline J.M. (2005). Mice heterozygous for a defect in mitochondrial trifunctional protein develop hepatic steatosis and insulin resistance. Gastroenterology.

[44] Li L., Lu D.Z., Li Y.M., Zhang X.Q., Zhou X.X., Jin X. (2014). Proteomic analysis of liver mitochondria from rats with nonalcoholic steatohepatitis. World J. Gastroenterol..

[45] Pepin M.E., Ha C.-M., Crossman D.K., Litovsky S.H., Varambally S., Barchue J.P., Pamboukian S.V., Diakos N.A., Drakos S.G., Pogwizd S.M. (2019). Genome-wide DNA methylation encodes cardiac transcriptional reprogramming in human ischemic heart failure. Lab. Investig..

[46] Doege H., Schürmann A., Bahrenberg G., Brauers A., Joost H.-G. (2000). GLUT8, a novel member of the sugar transport facilitator family with glucose transport activity. J. Biol. Chem..

[47] DeBosch B.J., Chen Z., Finck B.N., Chi M., Moley K.H. (2013). Glucose transporter-8 (GLUT8) mediates glucose intolerance and dyslipidemia in high-fructose diet-fed male mice. Mol. Endocrinol..

[48] Limesand S.W., Regnault T.R., Hay W.W. (2004). Characterization of glucose transporter 8 (GLUT8) in the ovine placenta of normal and growth restricted fetuses. Placenta.

[49] Janzen C., Lei M.Y., Jeong I.S.D., Ganguly A., Sullivan P., Paharkova V., Capodanno G., Nakamura H., Perry A., Shin B.C. (2018). Humanin (HN) and glucose transporter 8 (GLUT8) in pregnancies complicated by intrauterine growth restriction. PLoS ONE.

[50] Diaz M., Garde E., Lopez-Bermejo A., de Zegher F., Ibañez L. (2020). Differential DNA methylation profile in infants born small-for-gestational-age: Association with markers of adiposity and insulin resistance from birth to age 24 months. BMJ Open Diabetes Res. Care.

[51] Ollikainen M., Smith K.R., Joo E.J.-H., Ng H.K., Andronikos R., Novakovic B., Aziz N.K.A., Carlin J.B., Morley R., Saffery R. (2010). DNA methylation analysis of multiple tissues from newborn twins reveals both genetic and intrauterine components to variation in the human neonatal epigenome. Hum. Mol. Genet..

[52] Titus A.J., Gallimore R.M., Salas L.A., Christensen B.C. (2017). Cell-type deconvolution from DNA methylation: A review of recent applications. Hum. Mol. Genet..

