# Supplementary material for: DNA Methylation Signatures in Paired Placenta and Umbilical Cord Samples: Relationship with Maternal Pregestational Body Mass Index and Offspring Metabolic Outcomes
Source: Biomedicines. 2024 Jan 27;12(2):301. doi: 10.3390/biomedicines12020301 (PMC10886657; doi:10.3390/biomedicines12020301)
Supplement: Supplementary file 1 [file biomedicines-12-00301-s001.zip › Suppl Table 1_primers_210721.pdf]

Supplementary Table S1: Pyrosequencing primers and PCR conditions

Gene		Primers	Annealing temperature (ºC)	Fragment size (bp)
SLC2A8	Fw	GGGTTAGTTTGGAGTTTGTGTAGAG	56	115
	Rv	**BIOT**-CCCAACTACAACAAATAATCTCCAC		
	Seq	TTGTTTAGGTAGGATTGT		
HADHA	Fw	GTAAGGTAGTTTTGTTTTTGGAGTTTAG	56	224
	Rv	**BIOT**-AAATTTATATTAACATAATCCAACTCTCT		
	Seq	AAAATTTTATAAGATTATAGTTAG		

Primers were designed by the PyroMark Assay Design 2.0 software
