# Supplementary material for: DNA Methylation Signatures in Paired Placenta and Umbilical Cord Samples: Relationship with Maternal Pregestational Body Mass Index and Offspring Metabolic Outcomes
Source: Biomedicines. 2024 Jan 27;12(2):301. doi: 10.3390/biomedicines12020301 (PMC10886657; doi:10.3390/biomedicines12020301)
Supplement: Supplementary file 1 [file biomedicines-12-00301-s001.zip › Suppl Table 3_pyroseq and RTpcr.pdf]

Suppl Table S3: Levels of *HADHA* and *SLC2A8* methylation (by pyrosequencing) and expression (by RT-PCR) in placenta and umbilical cord

**PLACENTA**	**Methylation (%)**	**Expression ($2^{-\Delta CT}$)**
HADHA	5.70 ± 0.53	2.94 ± 0.12
SLC2A8	98.76 ± 0.23	0.76 ± 0.03
**UMBILICAL CORD**	**Methylation (%)**	**Expression ($2^{-\Delta CT}$)**
HADHA	14.8 6 ± 1.32	0.12 ± 0.01
SLC2A8	98.46 ± 0.32	0.01 ± 0.01
